# Accumulation of gene copy number variations during the early phase of free‐spawning abalone speciation

**DOI:** 10.1002/ece3.9816

**Published:** 2023-02-17

**Authors:** Shotaro Hirase, Masashi Sekino, Motoyuki Hara, Kiyoshi Kikuchi

**Affiliations:** ^1^ Fisheries Laboratory, Graduate School of Agricultural and Life Sciences The University of Tokyo Shizuoka Japan; ^2^ Bioinformatics and Biosciences Division, Fisheries Resources Institute Japan Fisheries Research and Education Agency Yokohama Japan; ^3^ Tohoku Ecosystem‐Associated Marine Sciences Tohoku University Sendai Japan

**Keywords:** ecological speciation, marine invertebrates, marine speciation, Western Pacific abalones, whole‐genome sequencing

## Abstract

The genetic basis of speciation in free‐spawning marine invertebrates is poorly understood. Although gene copy number variations (GCNVs) and nucleotide variations possibly trigger the speciation of these organisms, empirical evidence for such a hypothesis is limited. In this study, we searched for genomic signatures of GCNVs that may contribute to the speciation of Western Pacific abalone species. Whole‐genome sequencing data suggested the existence of significant amounts of GCNVs in closely related abalones, *Haliotis discus* and *H. madaka*, in the early phase of speciation. In addition, the degree of interspecies genetic differentiation in the genes where GCNVs were estimated was higher than that in other genes, suggesting that nucleotide divergence also accumulates in the genes with GCNVs. GCNVs in some genes were also detected in other related abalone species, suggesting that these GCNVs are derived from both ancestral and de novo mutations. Our findings suggest that GCNVs have been accumulated in the early phase of free‐spawning abalone speciation.

## Graphical abstract

Although gene copy number variations (GCNVs) and nucleotide variations possibly trigger the speciation of free‐spawning marine invertebrates, empirical evidence for such a hypothesis is limited. Our findings suggest that the accumulation of GCNVs contributes to the speciation of free‐spawning marine invertebrates during the early phase.

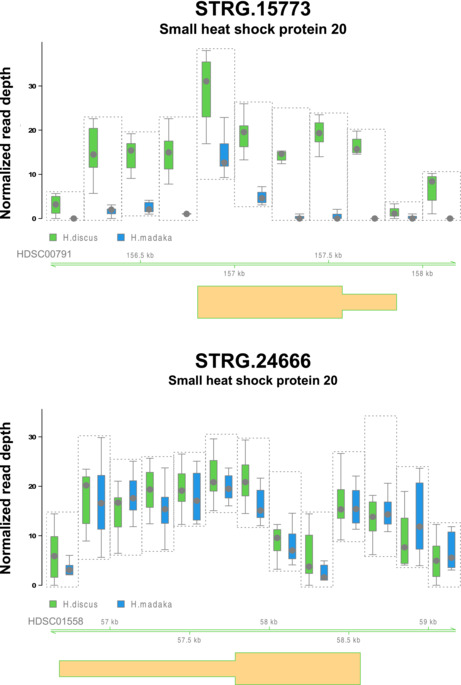

## 1 INTRODUCTION

A major goal in marine biology is understanding the genetic basis of speciation in free‐spawning marine invertebrates (Pogson, 2016). Genomic analyses based on single nucleotide polymorphism (SNP) loci have identified candidates for genetic variation that contributes to marine speciation (Hirase et al., 2021; Momigliano et al., 2017). However, a growing body of evidence suggests that gene copy number variations (GCNVs) also play an important role in the ecological divergence of various organisms (Castagnone‐Sereno et al., 2019; Hirase et al., 2014; Ishikawa et al., 2019; Pezer et al., 2015) including marine species (Dorant et al., 2020). Therefore, GCNVs, as well as nucleotide variations, may trigger speciation in the ocean; however, empirical evidence for these hypotheses is limited.

In the current study, we focus on GCNVs in the Western Pacific abalones, *Haliotis discus*, *H. madaka*, and *H. gigantea* (Ino, 1952). These three species are estimated to have diverged recently from North America and are genetically close to the North American abalones (Geiger & Groves, 1999; Hirase et al., 2021). Population genomic analyses of these species revealed that although the three species are genetically distinct, there is evidence of historical and ongoing gene flow among these species. The most closely related pair, *H. discus* and *H. madaka* (genome‐wide *F*_ST_ = 0.007), appears to occupy the early stages of the speciation continuum after the initial divergence of *H. gigantea* (Hirase et al., 2021). In the current study, we searched for GCNVs between *H. discus* and *H. madaka* based on whole‐genome sequencing (WGS) data. Our results showed the possibility of GCNVs accumulating during their speciation event. To further investigate whether the candidate GCNVs were derived from de novo or standing genetic variations, we examined GCNVs in *H. gigantea* and North American abalones that are expected to be genetically close to the Western Pacific abalones.

## 2 MATERIALS AND METHODS

### 2.1 Alignment of whole‐genome sequencing data

WGS data from the Western Pacific (eight *H. discus*, eight *H. madaka*, and six *H. gigantea*) and North American abalone species (three *H. rufescens*) were used. These were generated using Illumina HiSeq X Ten with the 150‐bp paired‐end protocol in our previous study (Hirase et al., 2021; Table A1 in Appendix A). The WGS data of five other North American abalone species (two individuals per species) were downloaded from the NCBI SRA (SRR7958743−SRR7958752; Masonbrink et al., 2019). The paired‐end reads were cleaned and aligned to the *H. discus  hannai* genome sequence (Nam et al., 2017) as described by Hirase et al. (2021). PCR duplicates were removed from constructed BAM files using SAMtools ver. 1.9 markdup (Li et al., 2009).

### 2.2 Identification of gene copy number variations

We compared the number of mapped reads for each gene, which was predicted in a previous study (Hirase et al., 2021), between *H. discus* and *H. madaka* using BAM files (Table A1 in Appendix A), and identified candidate GCNVs by referring to Hirase et al. (2014). If the number of mapped reads was significantly larger in one species, the gene would have been duplicated or multiplied specifically in that species. By contrast, if the numbers were significantly smaller, the gene would have been deleted, or its copy number would have decreased. Briefly, the number of mapped reads that overlapped with predicted gene regions (i.e., any exonic or intronic region) was counted using the featureCounts function of Subread ver. 1.4.6 (Liao et al., 2014). We removed genes onto which no reads were mapped in at least one individual, because an insufficient number of mapped reads may result in the detection of false GCNVs. We then searched for candidate GCNVs by detecting genes that showed significant differences in normalized read numbers between *H. discus* and *H. madaka* using the *edgeR* software package (Robinson et al., 2010) with a false discovery rate (FDR) < 0.01. For normalization with *edgeR*, the total number of mapped reads across the genome of each individual was used. To confirm that the number of identified GCNVs was significantly larger than expected by chance, we calculated an empirical *p*‐value based on a permutation test. In this test, we randomly reallocated eight *H. discus* and eight *H. madaka* individuals into two groups 10,000 times, performed the above analyses for each generated dataset, and obtained the null distribution of the numbers of GCNVs. Gene functions were annotated using BLASTP searches against the NCBI nonredundant (nr) protein database. We conducted BLASTN searches for candidate GCNVs against four North American abalone genomes (E‐value < 0.0000001): *H. fulgens* (halful_medaka.final_.fasta; https://abalone.dbgenome.org/downloads), *H. sorenseni* (h.sorensenigenomepilon3.pilon_.fasta; https://abalone.dbgenome.org/downloads), *H. rufescens* (Halruf.fasta; https://abalone.dbgenome.org/downloads; Masonbrink et al., 2019), and *H. cracherodii* (GCA_022045235; Orland et al., 2022).

To compare genetic differentiation between *H. discus* and *H. madaka* within and outside GCNVs, we calculated *F*_ST_ values for nonoverlapping 1 kb sliding windows based on SNP loci using VCFtools ver. 1.1.12 (Danecek et al., 2011). For this calculation, SNP information (a vcf file) that was obtained in Hirase et al. (2021) was used; In this study, SNPs were called for *H. discus* and *H. madaka*, *H. gigantea* using SAMtools mpileup (−Q 30) and bcftools, and filtered using VCFtools (‐‐minQ 20 ‐‐remove‐indels ‐‐maf 0.05 ‐‐max‐alleles 2 ‐‐minDP 6 ‐‐max‐missing‐count 1). This SNP information was used for the detection of three‐or‐more‐different allelic pairs later (*see* next section).

### 2.3 Detection of three‐or‐more‐different allelic pairs

**FIGURE A1:**
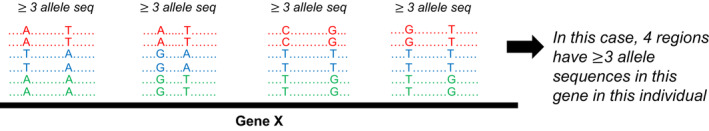
Schematic diagram of the method for identifying three‐or‐more‐different (≥3) allele sequences (haplotypes) in candidate gene copy number variation (GCNV) genes. The number of different allelic sequences was counted for each of the identified GCNVs by enumerating every pair of SNV positions that were located within the read length.

Three‐or‐more‐different allelic pairs (≥3 allele sequences) within a gene could be evidence of the GCNVs because ≥3 allele sequences of a gene cannot occur in a diploid genome (Hirase et al., 2014). Therefore, we examined whether ≥3 allele sequences were observed in the candidate GCNVs between *H. discus* and *H. madaka*, which were detected by read‐depth‐based analyses. The ≥3 allele sequences of candidate GCNVs were also identified in *H. gigantea*. In this analysis, we enumerated every pair of SNP positions, for each of the identified candidate GCNVs, that were located within 100 bp (within‐read‐length single nucleotide variation: SNP position pairs) using BAM files, the vcf file, and a custom Perl script (deposited in Dryad). Briefly, the number of different nucleotide pairs for each of the within‐read‐length SNP position pairs, which were supported by multiple (≥2) reads (by taking into account sequencing error) was counted. Then, we selected candidate GCNVs that were supported at least by one ≥3 allele sequence in at least three individuals from either species (Figure A1 in Appendix A). The ≥3 allele sequences in candidate GCNVs were also identified in North American abalone species. SNPs of North American abalones were called separately from those of the Western Pacific abalones, using the same method mentioned above, and filtered using VCFtools (−‐minDP 10 ‐‐remove‐indels ‐‐max‐missing‐count 0).

## 3 RESULTS

**FIGURE 1:**
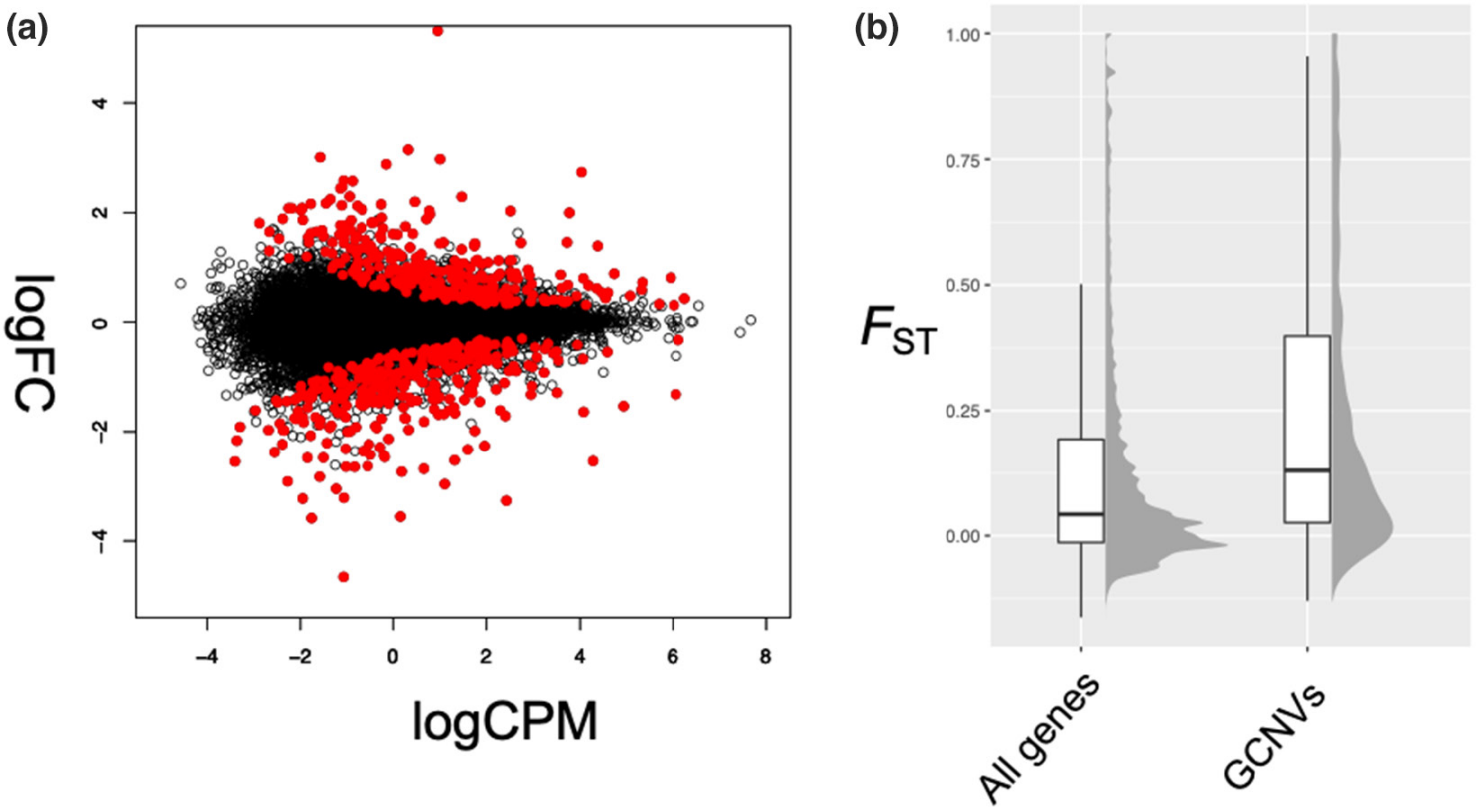
(a) MA plot showing the relationship between average concentration (logCPM) and fold‐change (logFC) across the genes. Each gene is represented by an open dot. Genes that showed significant differences in the number of mapped reads between *Haliotis discus* and *H. madaka* (FDR < 0.01) are colored in red. (b) Boxplot and half‐eye plot for sliding‐window *F*_ST_ values within all genes and those where gene copy number variations were expected.

Significant differences in the numbers of mapped reads between *H. discus* and *H. madaka* (FDR < 0.01) were observed for 627 genes (Figure 1a), and this number was significantly higher than expected by chance (*p* < .05). This suggests the accumulation of gene copy number differences between the two species. Among these genes, 328 genes were expected to have more copies in *H. discus* (called as HD‐increased GCNVs), and 299 genes were expected to have more copies in *H. madaka* (called as HM‐increased GCNVs). Next, we compared the sliding‐window *F*_ST_ within genes, where GCNVs were estimated, with those of all genes. The level of genetic differentiation in the genes where GCNVs were estimated was higher than that in all genes (Wilcoxon rank sum test; *p* < .05; Figure 1b).

**FIGURE 2:**
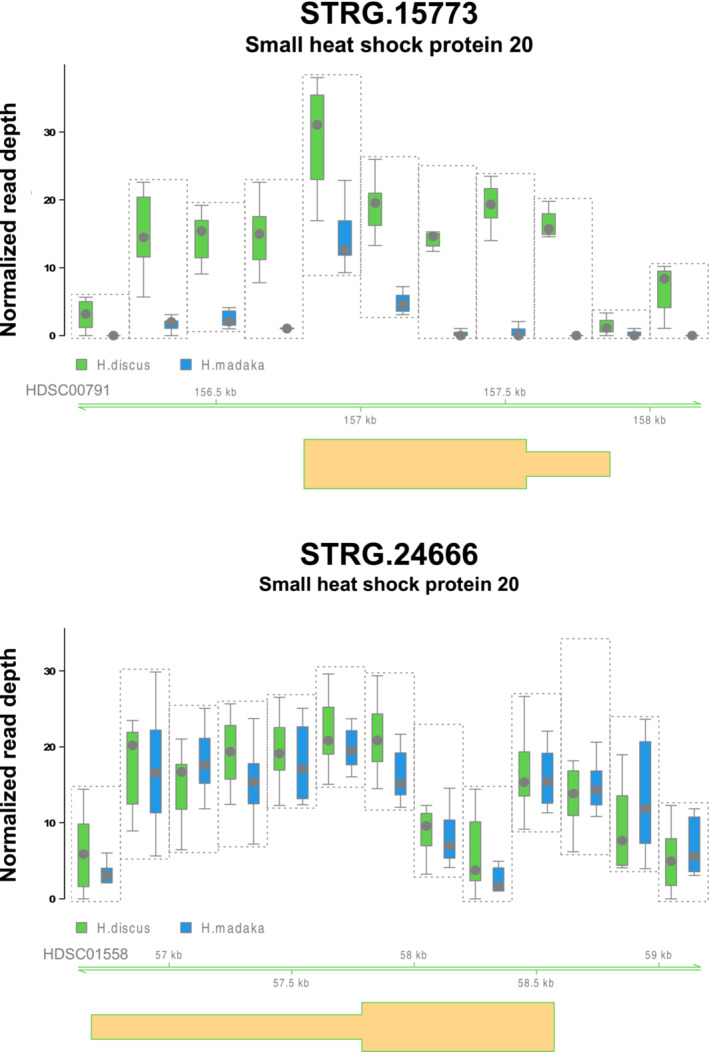
Distributions of normalized read depth in two genes possibly encoding small heat shock protein 20 (sHSP), STRG.15773 and STRG.24666. STRG.15773 is an HD‐increased gene copy number variation gene that was supported by the difference in the number of mapped reads between eight *Haliotis discus* and eight *H. madaka* individuals. Each boxplot represents the normalized read depth and average normalized read depth, respectively, of the mapped reads per 200‐bp nonoverlapping window for eight *H. discus* and eight *H. madaka* individuals. For normalization, the number of mapped read in each region was divided by the total number of mapped reads across the genome and multiplied by 10 million. Gene models are shown at the bottom of each panel.

Among the 328 HD‐increased GCNVs and 299 HM‐increased GCNVs, we focused on the top 10 HD‐increased GCNVs (Table A2 in Appendix A) and the top 10 HM‐increased GCNVs (Table A3 in Appendix A **)** and found that the top 10 HD‐increased GCNVs included the small heat shock protein 20 (*sHSP20*) gene (STRG15773). In the reference genome of *H. discus hannai* (Nam et al., 2017), two *sHsp20* genes were annotated as STRG.15773 (HDSC00791:156806‐157861) and STRG.24666 (HDSC01558:56682‐58572), which were predicted in our previous study (Hirase et al., 2021). Of the two genes, we detected HD‐increased GCNVs only in STRG15773 but not in STRG.24666 (Figure 2). Additionally, our BLASTN search suggested that there was only one *sHsp20* gene in the genome assemblies of the four North American abalones: *H. fulgens*, *H. sorenseni*, *H. rufescens*, and *H. cracherodii*.

**FIGURE 3:**
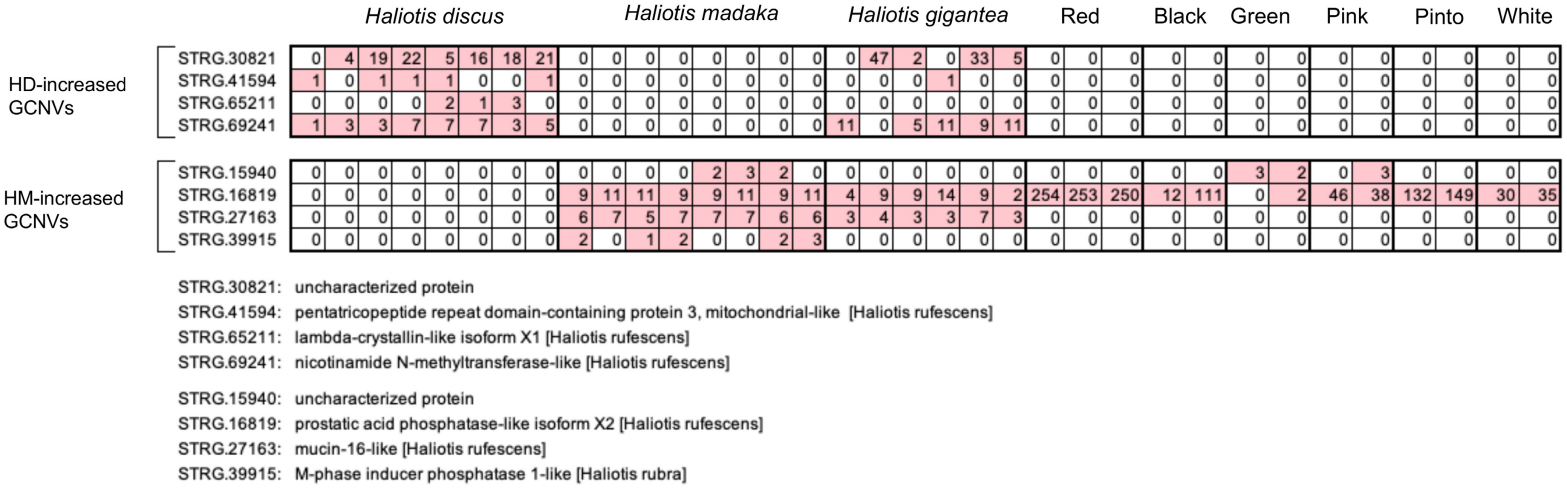
Four HD‐increased and four HM‐increased gene copy number variations that showed ≥3 allele sequences in at least three individuals. Each column represents an individual of each species, and the number in the box indicates the number of regions where ≥3 allele sequences were observed as shown in Figure A1 in Appendix A. Highlighted boxes show individuals that had ≥3 allele sequences in each gene. The ≥3 allele sequences were also identified in *Haliotis gigantea* and the six North American abalone species (Red: *H. rufescens*; Black: *H. cracherodii*; Green: *H. fulgens*; Pink: *H. corrugate*; Pinto: *H. kamtschatkana*; White: *H. sorenseni*).

Among the candidate GCNVs detected, four HD‐increased GCNVs had ≥3 allele sequences in at least three *H. discus* individuals and none in *H. madaka* individuals (Figure 3 **)**. Similarly, four HM‐increased GCNVs were supported by ≥3 allele sequences in at least three *H. madaka* individuals and no *H. discus* individuals (Figure 3). Among the eight genes where ≥3 allele sequences were detected in either *H. discus* or *H. madaka*, five genes also had ≥3 allele sequences in *H. gigantea* (Figure 3). Additionally, two of these five genes also had ≥3 allele sequences in the North American abalones (Figure 3 **)**. Although there were ≥3 allele sequences in one HM‐increased GCNV, STRG.16819, in all North American abalones, our BLASTN searches detected duplications/multiplications of this gene in the genome assembly of *H. sorenseni*, but not in those of the three North American abalone species, *H. fulgens*, *H. rufescens*, and *H. cracherodii*.

## 4 DISCUSSION

Gene copy number variations can cause organisms to inhabit new ecological niches (Ishikawa et al., 2019). We obtained genomic evidence of significant amounts of GCNVs between *H. discus* and *H. madaka*, which have different ecological niches and have recently speciated (Hirase et al., 2021). This result suggests that the accumulation of gene copy number differences is present in the early speciation stages of free‐spawning abalones. In addition, the degree of genetic differentiation in the genes where GCNVs were estimated was higher than that in other genes, consistent with previous observations that many CNVs were found in genes for which SNP‐based analyses detected signatures of positive selection (Feulner et al., 2013; Gokcumen et al., 2011; Hirase et al., 2014). This trend possibly suggests that nucleotide divergence also accumulates in the genes with GCNVs in abalones. Alternatively, there may be some biases in the *F*_ST_ estimates in the genes with GCNVs because alignments of multiple copies to one reference genome can cause the increased SNP variations in these genes (Feulner et al., 2013). This trend needs to be examined in more detail in the future.

Among the 627 candidate GCNVs detected, those in eight genes were confirmed by detecting ≥3 allele sequences in either *H. discus* or *H. madaka*. The method based on ≥3 allele sequences is not suitable for the detection of recently generated gene duplications, but instead robustly detects GCNVs of genetically distant species, because it does not depend on the efficiency of read mapping onto the reference genome, unlike depth‐based analysis (Nijkamp et al., 2012). We found that six of the eight genes had ≥3 allele sequences in *H. gigantea* and/or the North American abalone species. These results suggest that GCNVs between *H. discus* and *H. madaka*, which have recently diverged, are derived from both de novo (Zarrei et al., 2018) and standing genetic variations (Feulner et al., 2013). Within one gene (STRG.16819), we detected ≥3 allele sequences in more genomic regions in all the North American abalones species than in *H. discus* and *H. madaka*, which is consistent with the expectation that nucleotide mutations between standing duplicated genes have accumulated in the ancestral North American abalone species.

Our findings may suggest future issues regarding the genome assembly of abalones. Although the ≥3 allele sequences in one HM‐increased GCNV, the STRG.16819 gene, was detected in all North American abalone species, our BLASTN searches detected duplications/multiplications of genes in the genome assembly of *H. sorenseni*, but not in those of three North American abalone species, *H. fulgens*, *H. rufescens*, and *H. cracherodii*. Given that the genome assembly of *H. cracherodii* is a chromosome‐level assembly based on PacBio HiFi long‐reads (Orland et al., 2022), our results may imply that these GCNVs would have resulted from long segmental duplications/multiplications of genomic regions, which are longer than the PacBio HiFi long‐reads and remain unresolved (Vollger et al., 2019).

HD‐increased GCNVs included one of two genes that encode sHSP20 in the *H. discus hannai* genome. HSPs are the primary mitigators of environmental stress in various organisms (Chen et al., 2018) including abalones (Farcy et al., 2007; Huang et al., 2014; Kyeong et al., 2020). Variations in the copy number of *Hsp* genes have been found in diverse organisms. For example, in *Drosophila*, it has been suggested that *Hsp70* genes evolved into seven copies in thermotolerant species (Evgen'ev et al., 2004). *Hsp* genes comprise several families based on their molecular weights (Kampinga et al., 2009). Among these, *sHsp* genes are the smallest members of the *Hsp* superfamily. In *H. discus*, two *sHsp* genes, *sHsp26* and *sHsp20*, have been reported. The expression of these genes occurs in multiple tissues and is strongly affected by environmental stress (Park et al., 2008; Wan et al., 2012). In particular, *sHsp20* mRNA expression is rapidly elevated upon exposure to thermal, oxidative, and multiple toxic metal stresses (Wan et al., 2012), suggesting that this gene contributes to the adaptation of abalones to various environments. Since there was only one *sHsp20* in the genomes of the four North American abalones, the *sHsp20* gene duplication may have occurred after the speciation of Western Pacific abalone from the North American abalones (Geiger & Groves, 1999; Hirase et al., 2021), and that the copy number of STRG15773 increased specifically in *H. discus*. Previous gene expression analyses have indicated that the *sHsp20* gene is likely involved in abalone defenses against extreme environmental stress (Wan et al., 2012). Compared with other Western Pacific abalones, *H. discus* inhabits shallow depth zones where environmental fluctuations are more intense because of the influx of freshwater and the effects of varying water temperatures (Sinex, 1994). Therefore, it is possible that *sHsp20* is involved in the ecological adaptation of *H. discus*.

Among the eight GCNVs in which ≥3 allele sequences were detected, one HM‐increased GCNV was annotated as a mucin gene (Figure 3). Aquatic invertebrates protect the surfaces of their bodies, gills, and intestines with a mucus layer, which is composed of mucin glycoproteins (Bakshani et al., 2018). The mucus layer serves as an antimicrobial barrier and physical protective layer and has several physiological functions (Stabili, 2019). Copy number variations of mucin genes may affect the adaptive divergence between *H. discus* and *H. madaka*. The expression of this gene has been reported to respond to thermal stress in hybrids of *H. discus hannai* and *H. gigantea* (Xiao et al., 2021). Given that mucin genes belong to multigenic families (Desseyn et al., 1997), this finding is consistent with the idea that GCNVs of multigenic family genes are more likely to occur than those of single‐copy genes (Hirase et al., 2014; Nguyen et al., 2006).

This study provides the first empirical data showing GCNVs in the early phase of marine invertebrate speciation and suggests that GCNVs accumulate in the early phase of marine invertebrate speciation. In addition, some GCNVs were detected in ancestral species, suggesting that GCNVs are derived from both ancestral and de novo mutations.

## AUTHOR CONTRIBUTIONS

**Shotaro Hirase:** Investigation (lead); methodology (lead); writing – original draft (lead). **Masashi Sekino:** Resources (supporting); writing – review and editing (supporting). **Motoyuki Hara:** Resources (lead). **Kiyoshi Kikuchi:** Supervision (lead); writing – review and editing (equal).

## FUNDING INFORMATION

This work was supported by the Japan Society for the Promotion of Science (KAKENHI 21580240, 17K19280, 22H00377).

## CONFLICT OF INTEREST STATEMENT

We declare no competing interests.

## Data Availability

The WGS data used in this study are provided in Table A1 in Appendix A. The perl script for detecting ≥3 allele sequences is deposited in the Dryad Digital Repository: https://datadryad.org/stash/share/WGpUYJzAGlXloIlShuopLT65WheRf1ra1veScjvF7vg.

## APPENDIX A

**TABLE A1:** Summary of BAM files that used for identifying gene copy number variations in abalones.

Sample	Accession number	Species	Depth	Coverage (≥5 depth)	Mapped_read
HDD_Iwafune02	DRR276800	*Haliotis discus discus*	8.95	0.447	90131141
HDD_Iwafune04	DRR276801	*Haliotis discus discus*	9.29	0.456	92970560
HDD_Shibushi01	DRR276798	*Haliotis discus discus*	8.06	0.412	80852568
HDD_Shibushi02	DRR276799	*Haliotis discus discus*	8.95	0.441	88559647
HDD_Goto01	DRR276794	*Haliotis discus discus*	9.39	0.451	89424345
HDD_Goto02	DRR276795	*Haliotis discus discus*	10.31	0.470	98078183
HDD_Izu01	DRR276796	*Haliotis discus discus*	8.69	0.431	88093824
HDD_Izu02	DRR276797	*Haliotis discus discus*	9.07	0.444	89655629
HM_Misaki01	DRR276780	*Haliotis madaka*	9.30	0.446	88899019
HM_Misaki14	DRR276781	*Haliotis madaka*	9.92	0.459	92831843
HM_Misaki22	DRR276806	*Haliotis madaka*	11.12	0.480	101515775
HM_Misaki23	DRR276807	*Haliotis madaka*	10.69	0.475	97172750
HM_Mugi06	DRR276802	*Haliotis madaka*	9.41	0.448	96155304
HM_Mugi07	DRR276803	*Haliotis madaka*	10.04	0.459	99682383
HM_Mugi08	DRR276804	*Haliotis madaka*	10.72	0.474	96993674
HM_Mugi09	DRR276805	*Haliotis madaka*	10.58	0.471	97057005
HG_Tsukumi01	DRR276810	*Haliotis gigantea*	10.49	0.439	98095031
HG_Tsukumi02	DRR276811	*Haliotis gigantea*	9.44	0.421	86327844
HG_Sado01	DRR276808	*Haliotis gigantea*	9.19	0.415	84306984
HG_Sado03	DRR276809	*Haliotis gigantea*	9.61	0.424	85917618
HG_Goto01	DRR276812	*Haliotis gigantea*	10.13	0.433	94554293
HG_Goto02	DRR276813	*Haliotis gigantea*	11.15	0.449	99878405
HR08	DRR276814	*Haliotis rufescens*	10.24	0.384	85679394
HR09	DRR276815	*Haliotis rufescens*	11.40	0.399	97400854
HR10	DRR276816	*Haliotis rufescens*	10.27	0.382	89689486
Black01	SRR7958745	*Haliotis cracherodii*	16.07	0.300	96336284
Black02	SRR7958746	*Haliotis cracherodii*	11.31	0.277	66930259
Green01	SRR7958751	*Haliotis fulgens*	13.39	0.272	79977247
Green02	SRR7958752	*Haliotis fulgens*	12.88	0.269	77150186
Pink01	SRR7958747	*Haliotis corrugata*	12.00	0.272	69409947
Pink02	SRR7958748	*Haliotis corrugata*	15.08	0.286	88385778
Pinto01	SRR7958749	*Haliotis kamtschatkana*	11.18	0.286	66467241
Pinto02	SRR7958750	*Haliotis kamtschatkana*	15.19	0.307	93488007
White01	SRR7958743	*Haliotis sorenseni*	13.91	0.303	98281227
White02	SRR7958744	*Haliotis sorenseni*	16.19	0.309	101403398

**TABLE A2:** The number of mapped reads to top 10 HD‐increased gene copy number variations that divided by the number of the total mapped reads and their results of *edgeR*.

Gene	Annotation (top hit in BLASTP search)	*Haliotis discus discus*								*Haliotis madaka*								logFC	logCPM	*p*‐Value	FDR
		Iwafune02	Iwafune04	Goto01	Goto02	Izu01	Izu02	Shibushi01	Shibushi02	Mugi06	Mugi07	Mugi08	Mugi09	Misaki01	Misaki14	Misaki22	Misaki23				
STRG.52479	no hit	547	658	607	615	560	531	493	575	126	125	141	142	111	124	131	131	−2.261073	1.9575352	1.68 E‐154	2.70 E‐150
STRG.412	no hit	1536	3129	2825	3458	3579	3044	2969	3111	631	570	532	460	482	561	512	646	−2.5385	4.2779636	1.48 E‐79	1.59 E‐75
STRG.40757	XP_046329565.1| suppressor of cytokine signaling 4‐like [*Haliotis rufescens*]	266	237	235	278	255	221	216	228	55	42	32	56	30	42	30	38	−2.679361	0.6583958	2.04 E‐78	1.64 E‐74
STRG.48051	XP_046330233.1| protein SMG7‐like isoform X3	94	42	93	80	89	82	57	94	6	2	5	2	2	4	3	2	−4.656724	−1.077323	1.78 E‐50	9.52 E‐47
STRG.76	XP_046574497.1|uncharacterized protein LOC124282527 [Haliotis rubra]	186	354	276	407	501	532	339	420	64	52	69	96	49	55	65	117	−2.523546	1.3167576	4.13 E‐35	1.47 E‐31
STRG.15773	AMX23358.1|Hsp20 [Haliotis discus hannai]	97	81	115	108	103	89	89	89	32	26	14	20	17	16	21	15	−2.363456	−0.599399	3.40 E‐34	9.93 E‐31
STRG.45657	XP_046577082.1| uncharacterized protein LOC124284989 isoform X2 [Haliotis rubra]	53	40	57	44	47	55	38	33	5	4	6	2	7	2	2	4	−3.584297	−1.762253	1.69 E‐33	4.51 E‐30
STRG.43991	XP_046342493.1|septin‐7‐like isoform X1	421	461	422	405	355	427	382	381	208	225	222	218	224	208	193	239	−1.013928	1.7741263	8.39 E‐30	2.07 E‐26
STRG.37869	XP_046343616.1| beta‐1,4‐glucuronyltransferase 1‐like [*Haliotis rufescens*]	48	87	69	87	72	82	80	69	10	6	4	18	14	6	8	2	−3.212914	−1.069843	1.70 E‐29	3.91 E‐26
STRG.58440	XP_046344022.1|histone‐lysine N‐methyltransferase EHMT2‐like isoform X3 [*Haliotis rufescens*]	374	343	300	548	378	437	239	384	198	141	156	133	146	147	119	165	−1.413937	1.5279937	3.22 E‐26	6.09 E‐23
Total mapped number		9 E+07	9.3 E+07	8.9 E+07	9.8 E+07	8.8 E+07	9 E+07	8.1 E+07	8.9 E+07	8.9 E+07	9.3 E+07	1 E+08	9.7 E+07	9.6 E+07	1 E+08	9.7 E+07	9.7 E+07				

**TABLE A3:** The number of mapped reads to top 10 HM‐increased gene copy number variations that divided by the number of the total mapped reads and their results of *edgeR*.

Gene	Annotation (top hit in BLASTP search)	*Haliotis discus discus*								*Haliotis madaka*								logFC	logCPM	*p*‐Value	FDR
		Iwafune02	Iwafune04	Goto01	Goto02	Izu01	Izu02	Shibushi01	Shibushi02	Mugi06	Mugi07	Mugi08	Mugi09	Misaki01	Misaki14	Misaki22	Misaki23				
STRG.7630	XP_046362257.1|sushi, von Willebrand factor type A, EGF, and pentraxin domain‐containing protein 1‐like isoform X1 [*Haliotis rufescens*]	408	400	382	422	345	365	329	396	2262	2781	3308	2790	2494	2658	2845	2775	2.7368	4.02998	1.17 E‐225	3.75 E‐221
STRG.936	XP_046378335.1| uncharacterized protein LOC124150379 [*Haliotis rufescens*]	1038	1106	1029	1262	961	817	990	1015	3220	2533	2948	2895	2685	2861	3153	2935	1.3909	4.37891	2.89 E‐62	1.86 E‐58
STRG.17419	XP_046378335.1| uncharacterized protein LOC124150379 [*Haliotis rufescens*]	166	233	178	359	185	149	182	158	625	628	1044	1083	712	786	1096	1081	2.028	2.51044	1.27 E‐37	5.82 E‐34
STRG.24061	XP_046344413.1| uncharacterized protein LOC124125179 [*Haliotis rufescens*]	187	177	155	163	155	153	168	127	417	431	469	487	375	415	516	659	1.4363	1.74836	1.65 E‐36	6.62 E‐33
STRG.32296	XP_046330173.1| telomere length regulation protein TEL2 homolog isoform X2 [*Haliotis rufescens*]	585	359	472	449	421	645	403	568	2204	2440	1637	2847	2786	1907	1393	1512	1.9967	3.7715	2.20 E‐34	7.07 E‐31
STRG.59252	XP_046339330.1|countin‐3‐like isoform X1 [*Haliotis rufescens*]	32	8	5	36	36	39	6	16	210	203	215	200	203	192	264	212	3.1475	0.32136	1.87 E‐28	4.00 E‐25
STRG.73643	XP_046351856.1|tyrosine‐protein phosphatase nonreceptor type 23‐like isoform X2 [*Haliotis rufescens*]	110	79	50	67	49	34	38	48	226	310	290	278	202	316	208	257	2.0302	0.77255	1.53 E‐26	3.07 E‐23
STRG.53292	XP_046373113.1|uncharacterized protein LOC124146723 [*Haliotis rufescens*]	2	3	6	4	6	8	8	11	44	43	54	66	53	59	69	38	3.0092	−1.578	1.88 E‐25	3.02 E‐22
STRG.45154	XP_046335256.1|fibroblast growth factor receptor 3‐like isoform X1 [*Haliotis rufescens*]	25	36	23	23	37	31	26	29	106	130	117	80	107	106	130	107	1.8233	−0.4006	3.64 E‐25	5.56 E‐22
STRG.61550	XP_046326147.1|procollagen‐lysine, 2‐oxoglutarate 5‐dioxygenase 1‐like isoform X2 [*Haliotis rufescens*]	143	137	156	169	148	158	142	165	331	296	397	269	332	372	408	328	1.0557	1.40385	4.04 E‐23	5.63 E‐20
Total mapped number		9 E+07	9.3 E+07	8.9 E+07	9.8 E+07	8.8 E+07	9 E+07	8.1 E+07	8.9 E+07	8.9 E+07	9.3 E+07	1 E+08	9.7 E+07	9.6 E+07	1 E+08	9.7 E+07	9.7 E+07				
